# Exceptional surface and bulk electronic structures in a topological insulator, Bi_2_Se_3_

**DOI:** 10.1038/srep17351

**Published:** 2015-12-08

**Authors:** Deepnarayan Biswas, Sangeeta Thakur, Geetha Balakrishnan, Kalobaran Maiti

**Affiliations:** 1 Department of Condensed Matter Physics and Materials’ Science, Tata Institute of Fundamental Research, Homi Bhabha Road, Colaba, Mumbai - 400 005, India; 2 Department of Physics, University of Warwick, Coventry, CV4 7AL, UK

## Abstract

The outstanding problem in topological insulators is the bulk metallicity underneath topologically ordered surface states and the appearance of Dirac point far away from the Fermi energy. Enormous efforts are being devoted to get the Dirac point at the Fermi level via exposure to foreign materials so that these materials can be used in technology and realize novel fundamental physics. Ironically, the conclusion of bulk metallicity in the electronic structure is essentially based on the angle resolved photoemission spectroscopy, a highly surface sensitive technique. Here, we employed *state-of-the-art* hard *x*-ray photoemission spectroscopy with judiciously chosen experiment geometry to delineate the bulk electronic structure of a topological insulator and a potential thermoelectric material, Bi_2_Se_3_. The results exhibit signature of insulating bulk electronic structure with tiny intensities at 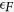 akin to defect/vacancy induced doped states in the semiconductors. The core level spectra exhibit intense plasmon peak associated to core level excitations manifesting the signature of coupling of electrons to the collective excitations, a possible case of plasmon-phonon coupling. In addition, a new loss feature appear in the core level spectra indicating presence of additional collective excitations in the system.

Recent experiments discovered that spin-orbit coupling can lead to new phases of quantum matter with highly nontrivial collective quantum effects. Topological insulators are one such realization, where surface states of a bulk insulator exhibit Dirac point quite distinct from graphene^1^,^2^. The surface states of a strong topological insulator are metallic with novel electromagnetic properties protected by the time reversal symmetry. One of the most studied topological insulators, Bi_2_Se_3_ is an archetypical thermoelectric material^3^. It forms in rhombohedral crystal structure with the space group 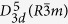. Extensive study of Bi_2_Se_3_ revealed topologically protected surface states possessing time reversal symmetry^1^,^2^,^4^,^5^,^6^,^7^. Ironically, the electronic structure exhibits numerous anomalies such as signature of metallic bulk, lattice termination dependence of the surface states^8^,^9^,^10^, significant sensitivity of the surface states to aging^4^,^5^,^6^,^7^, etc. Some observations indicate formation of two dimensional electron gas (2DEG) and Rashba states with time that has been explained employing impurity induced band bending scenario^6^,^11^,^12^.

Evidently, the behavior of the real materials is quite complex and far from idealistic scenario. While both the thermoelectricity and topological insulating behavior envisage bulk semiconductors as better candidates, experiments based on photoemission spectroscopy often exhibit bulk metallicity of these materials. One possibility of the absence of insulating bulk electronic structure in the experimental spectra could be the surface sensitivity of the angle resolved photoemission (ARPES) techniques^13^,^14^,^15^,^16^ employed to probe the electronic structure that manifests the metallicity of the subsurface layers, while the bulk is indeed insulating as predicted from band structure studies^17^,^18^. In order to reveal the bulk electronic structure, we employed high resolution hard *x*-ray photoemission spectroscopy^19^,^20^. The use of multiple photon sources, different experimental conditions and high energy resolution enabled us to identify the surface-bulk differences of the experimental spectral features. The valence band and core level spectra reveal exceptional scenario related to the topological order and thermoelectricity of this materials.

The escape depth, *λ* of the photoelectrons enhances with the increase in their kinetic energy, *KE* for *KE* ≥ 150 *eV*^13^,^14^. Therefore, photoemission with higher photon energy will be more sensitive to the bulk electronic structure. On the other hand, the enhancement of the electron emission angle with the surface normal at the same photon energy will enhance the surface sensitivity. The emission angle dependence is demonstrated in the schematic diagram in Fig. 1(a). We have exploited both the processes to identify the surface and bulk electronic structures. It is important to be noted that unchanged experimental geometry preserves the symmetry of photoemission as the light polarization direction remains protected, while the same photon energy helps to protect the photoemission cross section. This latter procedure is often helpful to probe the core level photoemission spectra, which are less sensitive to the polarization of incident light.

We investigate the Se 3*d* photoemission spectra measured at normal emission geometry with different photon energies. The spectra collected using hard *x*-ray (*hν* = 5947.9 eV, energy resolution = 150 meV) and Al *Kα* (*hν* = 1486.6 eV, energy resolution = 380 meV) energies are shown in Fig. 1(b). The hard *x*-ray photoemission (HXP) spectrum is convoluted with a suitable gaussian to compensate the resolution broadening of the Al *Kα* data, called as conventional *x*-ray photoemission (CXP) in this paper. The spectra are normalized by the intensity at the 3*d*_5/2_ peak. Two distinct peaks are observed corresponding to the spin-orbit split 3*d*_5/2_ and 3*d*_3/2_ signals. The intensity ratio of the spin-orbit split features depends on the ratio of their multiplicity, 2*j* + 1 (*j* = *l* + *s*; *j, l* and *s* are total, orbital and spin angular momenta, respectively), which is 3:2 for 3*d*_5/2_ and 3*d*_3/2_ features. The experimental data reveal an apparent breakdown of such scenario exhibiting different intensity ratio at different photon energies and none of them matches with 3:2 intensity ratio. The relative intensity of the 3*d*_3/2_ feature seem to be higher in the CXP spectrum compared to that in HXP data. The reproducibility of such anomaly has been confirmed by collecting several sets of data with different surface sensitivities.

To probe this deviation quantitatively, we simulated the experimental spectra using a set of asymmetric peaks following earlier studies^21^ - the asymmetry comes due to low energy excitations across the Fermi level. Consideration of at least two sets of spin-orbit split features is found to capture the intensity scaling to their multiplicities at all the experimental conditions studied. Typical fit for three different experimental setups are shown in Fig. 2 exhibiting remarkable representation of the experimental spectra. The spin-orbit split 3*d*_5/2_ and 3*d*_3/2_ features appear around 53.3 eV and 54.1 eV binding energies, respectively; spin-orbit splitting = 0.8 eV. The second set of spectra appear at 0.23 eV higher binding energies. The relative intensities of the second set with respect to the first set is calculated by the ratio of the integrated area under the corresponding curves and found to be of about 0.21, 0.46 and 1.01 at HXP (10° emission), CXP (normal emission) and CXP (60° emission), respectively.

Photoemission spectral intensity, 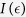 can be expressed as


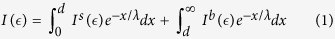


where, 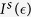 and 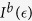 are the surface and bulk electronic structures, *d* represents the thickness of the surface layer and *λ* is the electron escape depth. Thus,


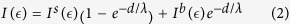


Therefore, the ratio of the surface and bulk contributions in the photoemission spectra can be expressed as


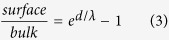


From the relative intensities of the surface and bulk contributions in the experimental spectra, we calculated the *d*/*λ* values to be 0.19, 0.378 and 0.698 employing the equation (3). While it is difficult to calculate *d* and *λ* independently, from our experience, we often found *λ* to be about 20 Å for the valence electrons in CXP measurements. Therefore, one can calculate *d* to be about 7.5 Å and *λ* to be about 39.5 Å, 19.8 Å and 10.7 Å for HXP, CXP (normal emission) and CXP (60° angled emission), respectively. These values of *λ* follows the energy dependence of 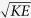 consistent with the universal curve as well as our previous experiments^13^,^14^. These results unambiguously link the higher binding energy features to the electronic structure of surface Se.

The analysis of the Se 3*d* spectra indicate a Se-termination of the sample upon cleaving. This is verified further by the angle resolved photoemission spectroscopy (ARPES). The high resolution ARPES data are shown in Fig. 3(a) and the corresponding energy distribution curves (EDC) are shown in Fig. 3(b). The experimental spectra exhibit a Dirac cone formed by the topologically ordered surface states with its apex at 0.3 eV binding energy confirming the Se-termination of the surface^8^.

The valence band (VB) spectra collected using various photon energies at normal emission are compared with the theoretically calculated spectral functions in Fig. 3(c,d). In order to calculate the spectral functions, we employed full potential linearized augmented plane wave (FLAPW) method as implemented in the Wien2k software^22^. The valence band is found to be constituted primarily by the Bi 6*p* and Se 4*p* partial density of states (PDOS). The CXP and HXP spectra were calculated considering photoionization cross-sections^23^ of the PDOS, their layer resolved contributions^24^ due to the inelastic scattering loss, and the resolution broadening. The theoretical results shown in Fig. 3(c,d) corroborate well with the experimental results; most of the features are reproduced in the calculations. However, it was necessary to shift the calculated results by 0.8 eV towards higher binding energies to match with the experimental results. While such rigid shift without spectral redistribution often appears due to electron correlation induced effect in semiconductors/insulators^25^, the correlation induced effects are weak in Bi_2_Se_3_. Here, the energy shift can be attributed to the defect/vacancy induced electron doping.

The lineshape of the CXP and HXP experimental spectra appears to be different with a relatively larger intensity around 2 eV in the CXP spectrum. This can be explained considering the difference in the photoionization cross-section at different photon energies as follows. The photoemission cross-section ratio of Se 4*p* and Bi 6*p* states 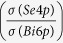 is about 5.5 times stronger in CXP than that at HXP^23^ suggesting enhancement of Se 4*p* contributions in CXP spectra. The enhancement of the intensity at 2 eV in the CXP spectrum indicates that it is primarily constituted by the Se *p* contributions. This is also manifested in the theoretical results; the constituent spectra obtained from Bi *p* and Se *p* partial DOS shown in the figure exhibit primary contribution from Se *p* states in the CXP valence band compared to the HXP valence band.

In addition to the above discussed cross-section induced effects in the photoemission spectra, there could be an influence from the change in surface sensitivity of the technique. This is investigated in Fig. 4(a), where the HXP valence band spectra taken at 10°, 45°, 60° emission angle and CXP data at normal emission are stacked together. In Fig. 4(b), we superimposed the HXP data collected at different emission angles and observe significant enhancement of intensity at 2 eV as the emission angle increases. Since the photon energy is same in this case, the cross-section induced effect is absent here and the change in intensities must be due to the change in surface sensitivity of the technique. Thus, the intensities at 2 eV represent surface electronic structure with dominant Se *p* contributions.

The intensities close to 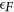 in all the experimental spectra appear to be nonexistent. This is unusual as the ARPES data revealed signature of significant bulk spectral intensity in addition to the metallic topologically ordered surface bands. In order to investigate this, we collected the data close to 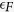 with good signal to noise ratio at different emission angles and different energies as shown in Fig. 4(c). The data reveal presence of finite intensity near 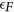; *it was possible to reveal such tiny contributions due to the employment of high energy resolution*. Interestingly, the relative intensity is somewhat higher for HXP at 10° emission than those found in Al *Kα* spectrum as well as at other emission angles of the HXP photoemission. This establishes presumably for the first time that the contributions at 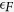 indeed possess dominant bulk character. Such weak but finite intensity of the charge carriers can arise due to defects and/or vacancies^26^ as observed in various other semiconducting systems^27^,^28^. All the spectra exhibit a dip at 0.3 eV below 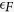, which corresponds to the energy position of the bulk band gap and the Dirac point of the surface bands as observed in the ARPES data shown in Fig. 3(a).

The above results establish that the energy bands close to the Fermi level possess bulk character arising presumably due to the defects/vacancy induced doping and the surface electronic structure consists of Dirac states with topological order. The large energy shift arises due to the population of the bulk conduction band by the doped states. Now, the question remains to be answered involves good thermoelectricity of these materials. The features in the core level photoemission spectra represent the excited states with a core hole present in the system. The CXP spectra of Bi 4*f* levels from uncleaved (dirty) and cleaved (clean) sample surface are superimposed over the HXP data in Fig. 5(a). Spin-orbit split Bi 4*f*_7/2_ and Bi 4*f*_5/2_ peaks appear around 158 eV and 163.3 eV binding energies with the spin-orbit splitting of 5.3 eV. Each spin-orbit split feature exhibit additional features at higher binding energies. The features, *α* at 159.4 eV and *α*′ at 164.65 eV are most prominent in the spectrum from dirty sample and absent in the spectrum from cleaved sample. These features are attributed to the 4*f* emission from Bi close to the sample surface bonded to impurity oxygens forming BiO_*x*_^29^,^30^.

The features, *β* and *β*′ appear at higher binding energies (160 eV and 165.7 eV), and are weak in the CXP spectrum. The intensity of these peaks become significantly strong in the HXP data. Some studies interpreted these peaks as Bi 4*f* satellites arising due to correlation induced effects^31^, whereas some other studies claimed them to be due to Se 3*p* contributions^32^,^33^. This puzzle can be resolved considering the spectral evolution with varied experimental conditions such as change in photon energy from Al *Kα* to hard *x*-ray photon energy and emission angles. We observed that the relative intensity of the features, *β* and *β*′ do not change with the change in emission angle with the same photon energy. This rules out the possibility of surface-bulk differences as an origin of the above mentioned intensity change.

The satellite-main peak description of the core level photoemission is as follows. The intense feature at 158 eV can be considered as the well screened feature, where the core hole is screened by a conduction/ligand electron. The feature at 160 eV can be described as the poorly screened feature/satellite. Such multiple features are observed to appear in correlated electron systems due to strong electron-electron Coulomb repulsion energy. This is unlikely to be applicable here as the electron correlation is found to be significantly weak in this system - the valence band is constituted by highly extended *p* states and is well described within the local density approximations. The intensity ratio of the screened and unscreened features corresponding to the photoemission from the same Bi 4*f* level is unlikely to be so significantly different in the CXP and HXP spectra shown in Fig. 5(a,b). Moreover, the energy separation between *β* and *β*′ is larger (~5.7 eV) than the spin-orbit splitting of Bi 4*f* level. Thus, it is unlikely that these features are satellite signal to Bi 4*f* photoemission.

The photoemission cross-section of Se 3*p* states relative to Bi 4*f* states 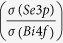 at Al *Kα* energy is significantly smaller than its value in HXP energy (CXP value is about 20% of its HXP value), which corroborates well with the change in intensity in the experimental results. Thus, the features, *β* and *β*′ can unambiguously be attributed to the Se 3*p* character. In order to verify this assertion further, we simulated the Bi 4*f* spectral region in a large energy window as shown in Fig. 5(b). Three distinct features are visible in the energy range 168–185 eV as shown by arrows and * in the inset of Fig. 5(b). We observe that all the features in the experimental spectra could be captured remarkably well along with their intensity scaling with the corresponding multiplicity ratio of Bi 4*f* and Se 3*p* photo-excitations. The simulated spectrum provides an wonderful representation of the experimental spectra as shown by solid line superimposed over the experimental spectrum.

The experimental spectrum and its simulated results in Fig. 5(b) reveal an interesting scenario. Two distinctly intense features appear at higher binding energies possessing similar energy separation and intensity ratio as that of the Bi 4*f* photoemission features due to the plasmon excitations in the final states of the 4*f* electron photoemission. These loss features are broad (FWHM 6 eV) compare to the main peaks (FWHM 0.4 eV), appear about 17 eV away from the main peak and can be attributed to the plasmon excitation induced loss features. Most interestingly, there is an additional set of two peaks structure (blue area plots) with intensity ratio and energy separation similar to the 4*f* spin-orbit split features. Distinct signature of the feature around 170 eV is demonstrated in the raw data - see the 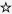 in the inset of Fig. 5(b). Therefore, these features (about 6.7 eV away from main peak) appear to be linked to the final state effects in the Bi 4*f* photo-excitations. The signature of these features could not be observed in the CXP spectra due to the appearance of Se Auger peaks in this energy range.

In order to ascertain the assignments of the origin of the features in Bi 4*f* spectra further, we investigate the HXP spectrum of Bi 4*d* level collected at 30 K shown in Fig. 6. Intense Bi 4*d*_5/2_ and 4*d*_3/2_ features appear around 441.1 eV and 464.8 eV binding energies, respectively. The features around 458.2 eV and 481.9 eV represent the plasmon loss features consistent with the scenario in Bi 4*f* spectrum. In addition, there are distinct signatures of two features around 447.5 eV and 471.4 eV. To disentangle the constituent features in the 4*d* spectrum, we fit the spectral function following the procedure similar to the case of 4*f* level. The results demonstrate the presence of the 447.5 and 471.4 eV features in addition to the plasmon loss features, which are quite similar to those appeared in the Bi 4*f* spectrum. The appearance of these features in all the core level spectra indicates that their origin involves the energy loss due to collective excitations in the materials.

Bi_2_Se_3_ has been considered to be a good candidate for thermoelectric devices. The *figure of merit* of a thermoelectric material can be expressed as 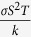, where *σ* is the electrical conductivity, *S* is the Seebeck coefficient, *T* is temperature and *κ* is the thermal conductivity. Evidently, a good electrical conductor and a bad thermal conductor (small gap semiconductors) with high atomic number, *Z* are expected to be good materials for such behavior. While stoichiometric Bi_2_Se_3_ is expected to be a band insulator^8^,^10^,^17^,^18^, the experiments show metallicity in their electronic structure^1^, which is now established to be due to the defect/vacancy induced electron doping present in these materials^26^. Thus, the conduction band gets populated by such effects as observed in the ARPES spectra shown in Fig. 3, making electron pockets in the Fermi surface around Γ-point.

The core level spectra exhibit signature of strong plasmon excitations, which involve collective excitations of charge-density oscillation. The additional features discovered between the main peak and the plasmon peak in both Bi 4*f* and 4*d* core level spectra correspond to some collective excitation induced loss features. The presence of strong plasmon mode in the core level spectra manifests signature of coupling of electrons to the collective excitations and hence the possibility of significant plasmon-phonon coupling^34^,^35^. Signature of phonon excitations has been observed in photoemission spectra in earlier studies^36^. This could be the reason for strong phonon scattering in this material leading to an enhancement of the figure of merit in their thermoelectric property. Clearly, more studies are required to understand the origin of such spectral functions.

In summary, we have studied the detailed electronic structure of Bi_2_Se_3_ employing high resolution hard *x*-ray photoemission spectroscopy. The valence band could be described well within the generalized gradient approximation (GGA) method indicating weak influence from electron correlation. High energy resolution enabled us to identify tiny features close to the Fermi level forming the topologically ordered surface states and the bulk bands arising due to defects/vacancies in the system. These results establish that Bi_2_Se_3_ is basically a semiconductor with impurity states populating the bottom of the conduction band. Thus, the appearance of the Dirac point to the Fermi level depends primarily on the concentration of the defects/impurities in the material rather than deposition of donor/acceptor impurities. The core level spectra exhibit strong plasmon excitation peaks indicating signature of the coupling of electrons to the collective excitations. In addition, we discover a new loss feature appearing in between the main peak and plasmon satellite suggesting further the link of the electron excitations to various collective modes in the system - further studies are required to find the origin of such features.

## Method

High quality single crystal samples were prepared by the modified Bridgman method and *x*-ray laue diffraction was used to ascertain the crystalline quality. Hard *x*-ray photoemission (HXP) measurements were carried out at the P09 beamline of Petra III, Hamburg, Germany using 5947.9 eV photon energy and Phoibos analyzer. The energy resolution during the measurements was set to 150 meV. Conventional *x*-ray photoemission (CXP) measurements were carried out at the electron spectroscopy laboratory of TIFR, India using monochromatic Al *Kα* photon source (*hν* = 1486.6 eV) and R4000 WAL electron analyzer from Gammadata Scienta. The energy resolution for the CXP measurements were set to 380 meV to get good signal to noise ratio. Angle resolved photoelectron spectroscopic (ARPES) measurements were carried out using a monochromatic He I (*hν* = 21.2 eV) source with an energy resolution of 10 meV and angle resolution of 0.1°. The CXP and ARPES measurements were carried out on cleaved surfaces at a base pressure better than 1 × 10^−10^ torr. The vacuum during HXP measurements was 2 × 10^−10^ torr. The *in situ* cleaving was done using a top-post removal method. The experiment temperature of 30 K was achieved by an open cycle liquid He cryostat.

Electronic structure calculations were carried out using full potential linearized augmented plane wave (FLAPW) method^22^ and generalized gradient approximations (GGA)^37^. In order to calculate the layer resolved density of states, we carried out a slab calculation, where we considered a Se terminated slab containing 5 quintuple layers (Se-Bi-Se-Bi-Se layers) in symmetric geometry. The spin orbit coupling was included as a perturbation. The required lattice parameters were taken from the literature^38^.

In order to compare the experimental and theoretical results, we calculated the spectral functions from the partial density of states. The procedure adopted is as follows - first, the partial density of states from each of the layers constituting the valence band were multiplied by the corresponding photoionization cross-sections^23^ separately. The contribution of these layer-resolved results to the valence band are calculated considering the escape depth for CXP and HXP measurements from each of the layers. All these contributions are then convoluted by the Fermi-Dirac function and the resolution broadening function (FWHM = 380 meV for CXP and 150 meV for HXP data).

## Additional Information

**How to cite this article**: Biswas, D. *et al.* Exceptional surface and bulk electronic structures in a topological insulator, Bi_2_Se_3_. *Sci. Rep.* **5**, 17351; doi: 10.1038/srep17351 (2015).

## Figures and Tables

**Figure 1:**
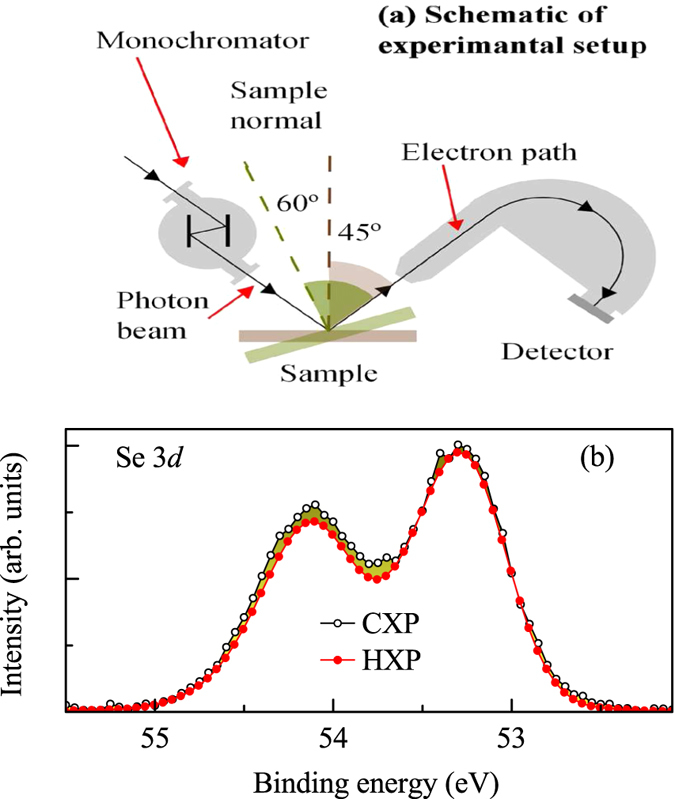
(**a**) A schematic of the experimental setup showing photon source, electron detector and sample for different electron emission angles. (**b**) CXP (Al *Kα*) and HXP spectra of Se 3*d* level exhibiting unusual change in spin-orbit split features.

**Figure 2:**
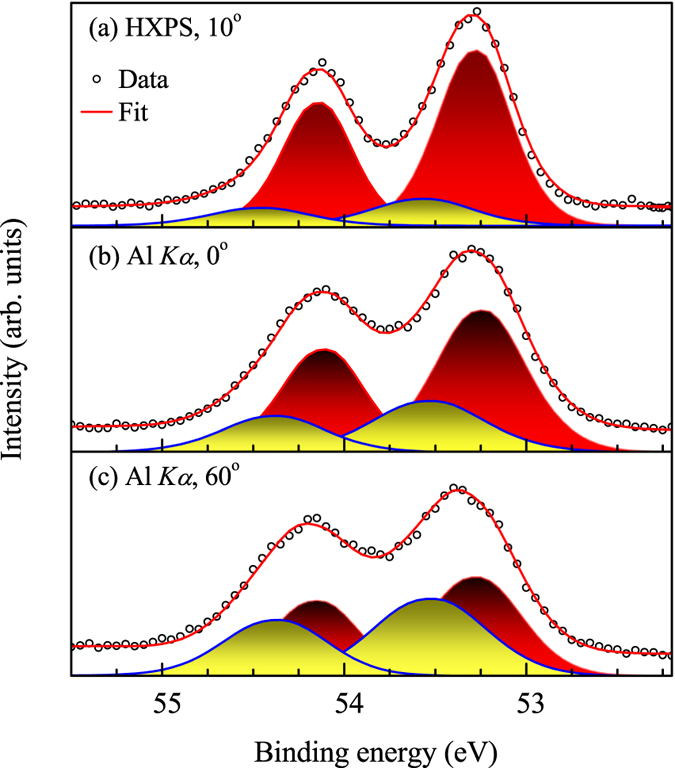
(**a**) HXP spectrum at 10° emission, CXP spectra at (**b**) normal emission & (**c**) 60° emission. Open circles represent experimental data and the superimposed lines represent the fit. The shaded regions show the component peaks simulating the experimental spectra.

**Figure 3:**
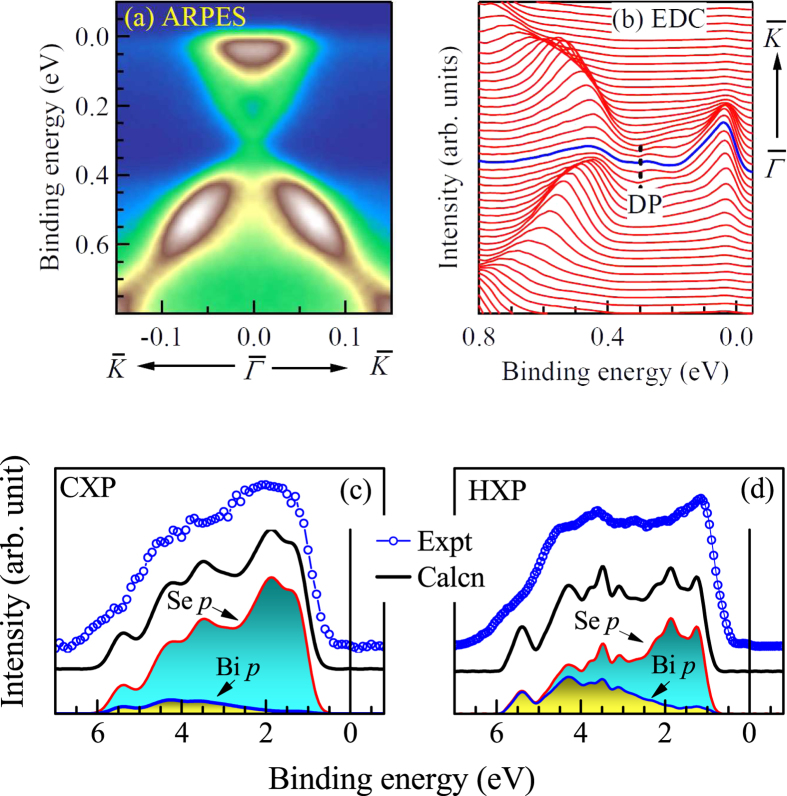
(**a**) ARPES data exhibiting the Dirac cone (DC) and (**b**) the corresponding energy distribution curves (EDCs). (**c**) CXP and (**d**) HXP valence band spectra along with corresponding theoretical spectral functions exhibiting a good representation. The component Bi *p* and Se *p* contributions are also shown by area plots.

**Figure 4:**
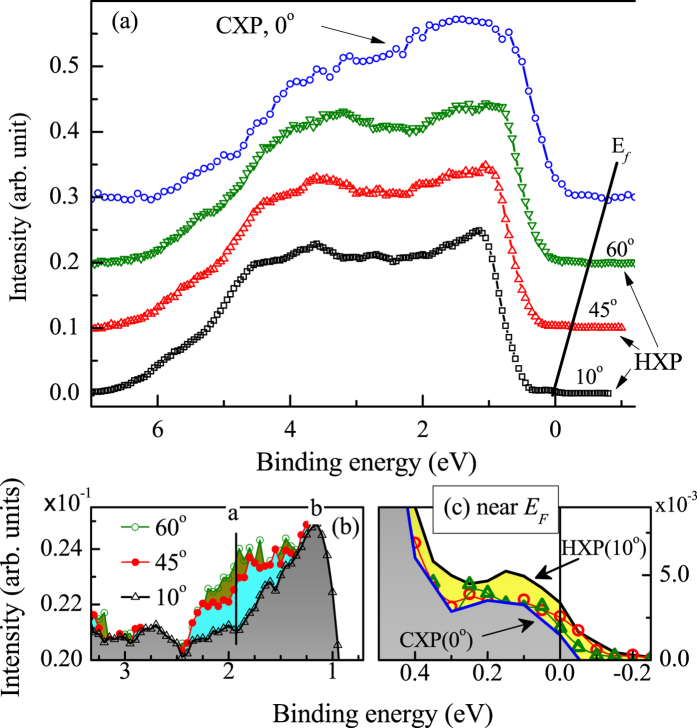
(**a**) HXP data at 10°, 45° & 60° emission angle, and CXP data at normal emission. (**b**,**c**) show the superimposed spectra shown in (**a**) at an enhanced intensity scale near 1.5 eV and near Fermi level, respectively.

**Figure 5:**
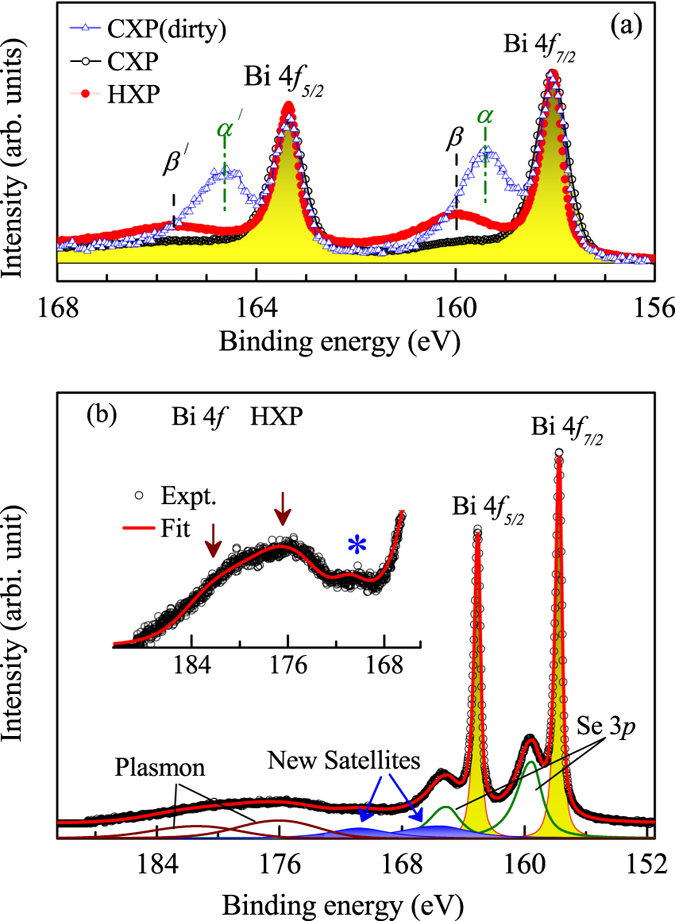
(**a**) CXP and HXP spectra of Bi 4*f* level collected from cleaved and uncleaved (dirty) sample surface. (**b**) Bi 4*f* HXP spectrum (symbols) and the fitted spectrum (line). Area plots represent the component features constituting the spectral function. The inset shows the satellites (marked by arrows and *) in an expanded intensity scales.

**Figure 6:**
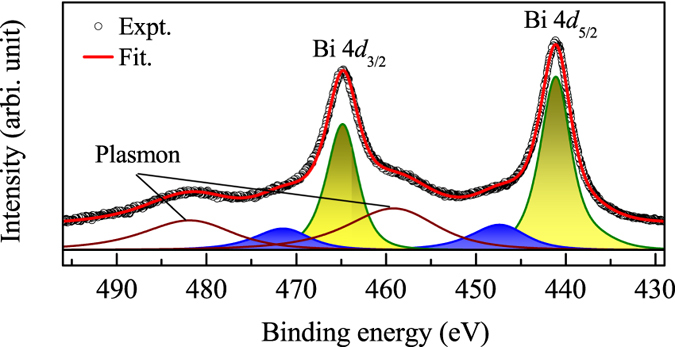
Bi 4*d* HXP spectrum. The symbols are the experimental result and the line represents the fit. The shaded regions represent the features constituting the experimental features.
